# Vernalization-Repression of Arabidopsis *FLC* Requires Promoter Sequences but Not Antisense Transcripts

**DOI:** 10.1371/journal.pone.0021513

**Published:** 2011-06-21

**Authors:** Chris A. Helliwell, Masumi Robertson, E. Jean Finnegan, Diana M. Buzas, Elizabeth S. Dennis

**Affiliations:** CSIRO Plant Industry, Canberra, Australia; Instituto de Biología Molecular y Celular de Plantas, Spain

## Abstract

The repression of Arabidopsis *FLC* expression by vernalization (extended cold) has become a model for understanding polycomb-associated epigenetic regulation in plants. Antisense and sense non-coding RNAs have been respectively implicated in initiation and maintenance of *FLC* repression by vernalization. We show that the promoter and first exon of the *FLC* gene are sufficient to initiate repression during vernalization; this initial repression of *FLC* does not require antisense transcription. Long-term maintenance of *FLC* repression requires additional regions of the gene body, including those encoding sense non-coding transcripts.

## Introduction

The Arabidopsis *FLC* gene is a repressor of flowering that confers a requirement for vernalization (a long period of cold, such as occurs during winter) to promote flowering in spring [1], [2]. *FLC* encodes a MADS box protein that binds to and represses expression of the floral promoting genes *FT* and *SOC1* [3], [4] in addition to regulating other developmental pathways [5]. Vernalization leads to the stable repression of *FLC* by a plant homeodomain-polycomb repressive complex 2 (PHD-PRC2) mechanism [6], [7] that results in increased abundance of H3K27me3 at the *FLC* locus. Detailed studies of the timing of changes in *FLC* mRNA expression and H3K27me3 levels showed that transcription of *FLC* is repressed and H3K27me3 increases at a region around the transcription start site during the cold [8]. After plants are returned to warm conditions, the level of H3K27me3 increases across the gene body and this is associated with the maintenance of repression of *FLC* transcription [8]. Plants with reduced PRC2 function have increased *FLC* expression and reduced repression of *FLC* after vernalization [6], [9], suggesting that the presence of H3K27me3 at the *FLC* locus is important in down-regulating its expression pre- and post-vernalization. Experiments to define the parts of the *FLC* gene mediating the different phases of the vernalization response showed that the promoter and first exon are sufficient to confer repression of a reporter gene during vernalization, but maintenance of repression after return to warm conditions requires the first intron in addition to the promoter and exon 1 [10]. An *FLC* gene fragment including approximately 1.8 kb of *FLC* intron 1 and the remainder of the 3′ end of the gene recruits PRC2 and H3K27me3 in the absence of transcription [11]; it is suggested that this property is responsible for maintaining *FLC* repression after vernalization.

**Figure 1:**
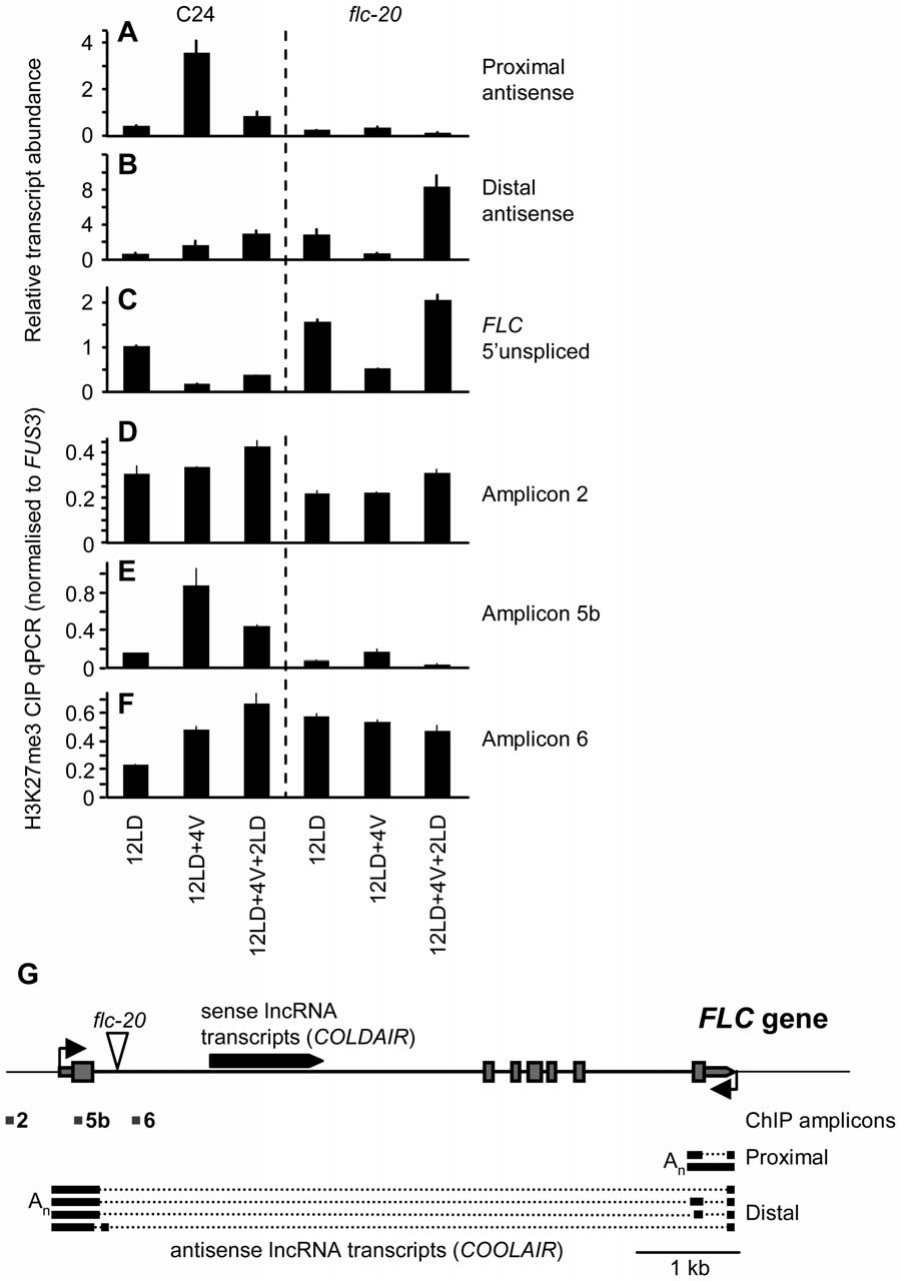
FLC is transiently repressed by extended cold in the *flc-20* mutant. A, B and C) qPCR quantification of proximal antisense (A), distal antisense (B) [28], and *FLC* 5′ unspliced transcript (C) is shown for C24 and *flc-20* for plants grown for 12 long days in warm conditions (12LD), 12 days in warm and transferred to cold conditions for 4 weeks (12LD+4V) or cold treated then returned to warm conditions for 2 days (12LD+4V+2LD). D, E and F) H3K27me3 ChIP-qPCR for amplicon 2 (D), amplicon 5b (E) and amplicon 6 (F) in C24 and *flc-20*, on same plant samples used in A–C. G) Diagram of the *FLC* gene and associated lncRNAs. Exons of the sense transcript are shown boxed, the exons of mature antisense transcripts [16] are shown in bold, introns in antisense transcripts are shown as dotted lines. Transcription starts indicated by arrows, triangle marks *Ds* insertion in *flc-20*. The *Ds* inserted in intron 1 of *FLC* in *flc-20* contains a GUS reporter gene and *nptII* resistance gene [21]. It is likely that the transcript detected with the distal primers in *flc-20* originates from within the *Ds* insertion as no PCR product is obtained using a primer set that spans the large intron in the distal antisense transcripts [18].

A complex array of non-coding RNAs is transcribed from eukaryotic genomes, the majority of which are of unknown function. Some long non-coding RNAs (lncRNAs) associate with and target protein complexes to regulate gene expression [12], [13]; these include the well-characterised lncRNA, *HOTAIR*, which targets PRC2 to the *HoxD* locus, and is associated with *HoxD* silencing in humans [14], [15]. Study of lncRNAs in plants is still in its infancy, but two classes of lncRNAs produced from the *FLC* locus have been identified. The *COOLAIR* antisense transcripts originate from a promoter adjacent to the *FLC* 3′ untranslated region and consist of two classes, terminating at proximal or distal sites (**Figure 1**) [16], [17], [18]. Antisense transcript levels increase during vernalization prior to the decrease of *FLC* mRNA abundance. *COOLAIR* promoter-driven antisense transcription of a reporter gene confers transient cold-induced repression [19]. This led to the suggestion that induction of antisense transcription is an early event in the mechanism causing vernalization-induced repression of *FLC*, acting upstream of PHD-PRC2 [19]. The second class of lncRNAs are sense transcripts (termed *COLDAIR*) originating from a region within the first intron of *FLC* [20]. The *COLDAIR* transcript has been shown to interact with PRC2 and its abundance also increases during vernalization. Reduction of *COLDAIR* transcript levels by RNAi showed that it is not required for the initial repression of *FLC* but is required for subsequent maintenance of repression.

As there are apparent contradictions in the proposed role of the *COOLAIR* antisense transcripts in the initial vernalization-induced repression of *FLC* and the results of reporter gene studies we used *FLC* insertion mutants to test the role of these transcripts in *FLC* repression.

## Results and Discussion

As the *FLC* promoter and first intron are required for the stable vernalization-mediated down-regulation of a reporter gene we tested the effect of a *Ds* insertion of approximately 6 kb (*flc-20*) that separates these regions in the endogenous *FLC* gene [21]. Using the abundance of *FLC* 5′ unspliced transcript as an approximation of transcription rate [18] we showed that vernalization leads to a reduction of transcription in the *flc-20* mutant that was not maintained after plants were returned to warm conditions (**Figure 1A**). The results of physically separating the *FLC* promoter and first intron thus mirror those for reporter constructs showing that the intron and promoter together are required for the stable repression of *FLC* expression by vernalization. Examination of antisense transcripts in these lines showed that transcription from the *COOLAIR* promoter was greatly reduced in *flc-20* and was only weakly induced during the cold compared to the C24 control (**Figures 1B and 1C**). An antisense transcript was detected 5′ of the Ds insertion in *flc-20* that we speculate originates from within the *Ds* element; in contrast to the situation in C24 this transcript was repressed by cold. There is an increase of H3K27me3 across the first exon of *FLC* (amplicon 5b) in both C24 and *flc-20* at the end of cold, but this increase is not maintained in *flc-20* after return to warm temperatures (**Figure 1E**), suggesting that sequences in intron 1 are required to maintain a repressed chromatin state in this region. Sites 3′ of the *Ds* element are marked with H3K27me3 before vernalization [11] and there is no increase following vernalization (**Figures 1F** **and S1**).

**Figure 2:**
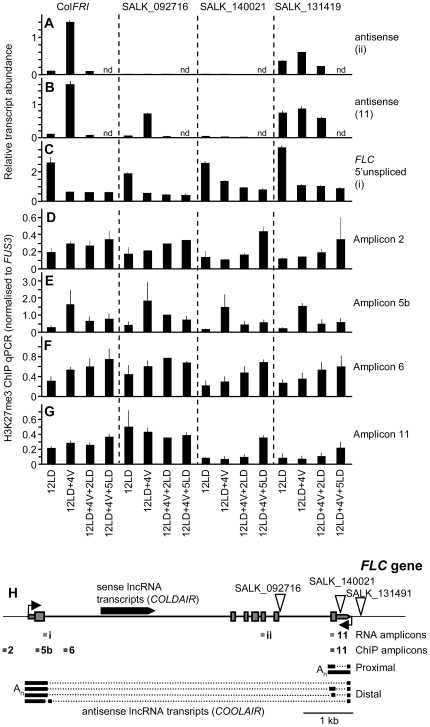
*COOLAIR* lncRNAs are not required for cold-repression of *FLC*. A, B and C) qPCR quantification of antisense (A, B), and *FLC* 5′ unspliced transcript (C) is shown for Col*FRI*, SALK_092716, SALK_140021 and SALK_131491 for plants grown for 12 long days in warm conditions (12LD), 12 days in warm and transferred to cold conditions for 4 weeks (12LD+4V) or cold treated then returned to warm conditions for 2 or 5 days (12LD+4V+2LD, 12LD+4V+5LD). D, E, F and G) H3K27me3 ChIP-qPCR for amplicon 2 (D), amplicon 5b (E), amplicon 6 (F) and amplicon 11 (G) in Col*FRI* and T-DNA insertion lines. H) Diagram of *FLC* gene, triangles mark T-DNA insertion sites. n.d.; not determined.

The data from the *flc-20* mutant together with previous data from *FLC* reporter constructs raised the question of whether antisense transcription from the *COOLAIR* promoter is a requirement for vernalization-induced repression of *FLC*. To test this we carried out further experiments with T-DNA insertions close to the 3′ end of the *FLC* gene that disrupt the distal (SALK_092716) or both proximal and distal (SALK_140021) antisense transcripts (**Figures 2A and 2B**). The T-DNA lines were crossed to Col*FRI* [22] to introduce an active allele of *FRI* to activate *FLC* transcription; plants homozygous for the T-DNA insertion and the active *FRI* allele were used in subsequent experiments. Sense transcription of *FLC* is stably repressed by cold when the distal antisense or both distal and proximal antisense transcripts are absent (**Figure 2C**). In addition, plants in which antisense transcript levels do not increase during cold (SALK_131491) showed normal repression of *FLC*. The changes in H3K27me3 in the T-DNA insertion lines mirrored those of the wildtype *FLC* allele in Col*FRI* (**Figures 2D–G** **and S2**), with the exception of regions 3′ of the SALK_092716 insertion which had high H3K27me3 under all conditions as seen previously for non-transcribed parts of *FLC* [11]. The remaining two insertion lines, SALK_140021 and SALK_131419, generally had lower levels of H3K27me3 at amplicon 11 than wildtype plants (**Figure 2G**).

These data show that the production of *COOLAIR* transcripts is not an essential component of vernalization-induced repression of *FLC*. The observations presented here for these SALK lines are in agreement with previous results showing that sequences consisting of the promoter, exon 1 and intron 1 but not the *COOLAIR* promoter, are sufficient to confer stable repression on a reporter gene [10]. While the *COOLAIR* lncRNAs may play a redundant role in regulating *FLC* expression, our data shows that they are not required for the vernalization response.

Our data are consistent with the *COLDAIR* lncRNA acting to maintain repression of *FLC* by recruiting the PRC2 machinery [23]. None of the T-DNA insertions tested interrupt the *COLDAIR* transcript and all show maintenance of repression after vernalization.

**Figure 3:**
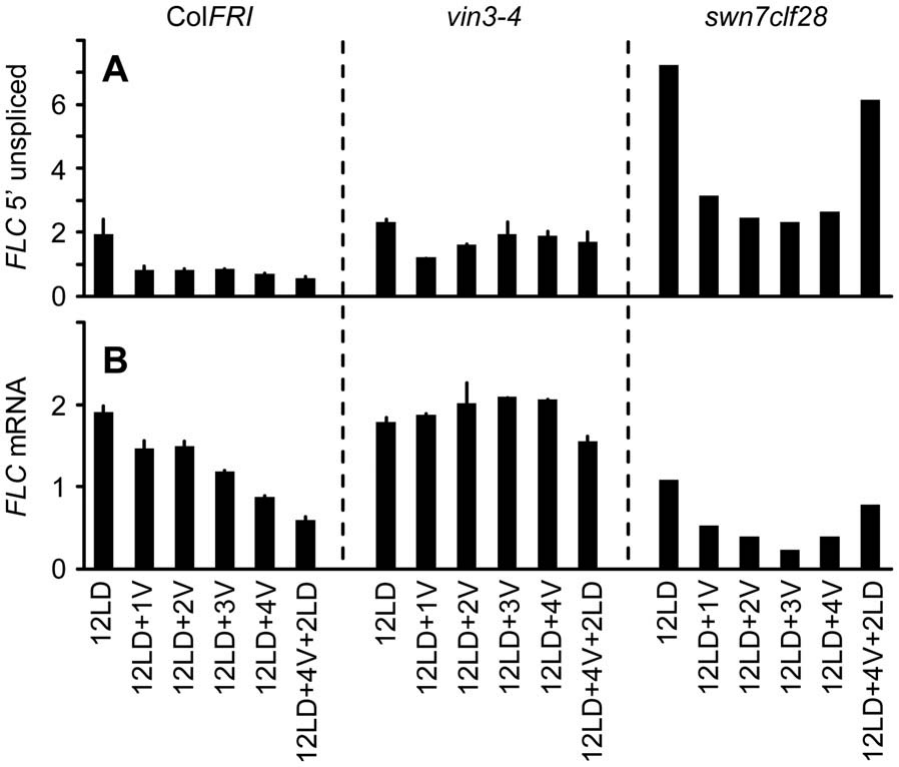
Initial repression of *FLC* transcription is not dependent on VIN3 or PRC2. A and B) qPCR of *FLC* 5′ unspliced (A) and mature *FLC* mRNA (B)in Col*FRI*, *vin3-4* and *swn7clf28* plants grown for 12 long days in warm conditions (12LD), 12 days in warm and transferred to cold conditions for 1–4 weeks (12LD+1V, 2V, 3V, 4V) or cold treated then returned to warm conditions for 2 days (12LD+4V+2LD).

To further investigate factors required for the initial repression of *FLC*, we measured 5′ unspliced *FLC* transcripts in the *swn7clf28* double mutant and in the *vin3-4* mutant. *CLF* and *SWN* encode histone methyl transferases components of PRC2; loss of function of these genes leads to a genome-wide loss of H3K27me3 [24]. The *swn7clf28* plants have increased 5′ unspliced *FLC* transcript in non-vernalized plants and show a similar fold-repression as Col*FRI* during 4 weeks of cold exposure (**Figure 3A**). However this repression is not maintained after plants are returned to warm conditions in agreement with previous reports that the PRC2 complex functions in the maintenance of repression rather than initiation [9], [25]. The *vin3-4* mutant showed a different pattern of *FLC* repression with an initial reduction in transcription after 1 week in the cold that was not maintained during subsequent weeks in the cold (**Figure 3A**). Previous reports showed no decrease in mature *FLC* mRNA [26] in *vin3* during vernalization which we confirmed (**Figure 3B**). These data suggest that although VIN3 interacts with the PRC2 complex it may have an additional role in establishing repression of *FLC* before the addition of H3K27me3 to *FLC* by PRC2.

The mechanism of the initial repression of *FLC* remains unknown, with none of the genes or other factors identified as being involved in the vernalization-induced repression of *FLC* to date being required for the initial repression by cold. Our data suggest that under our growth conditions the reduction of *FLC* transcription activity is saturated after 1 week of cold. However this treatment is not sufficient to saturate the vernalization response in Col*FRI* suggesting that subsequent events in the cold are required to establish a repressed chromatin state at *FLC* that is reinforced by the addition of H3K27me3 across the whole gene body after return to warm growing conditions.

## Materials and Methods

Salk insertion mutants were obtained from the Arabidopsis Stock Center (www.arabidopsis.org) and crossed with Col*FRI*, a Col line with an active *FRI* allele [22]. The presence of an active *FRI* allele activates expression of *FLC*. PCR was used to identify F2 plants in which the active *FRI* allele and the T-DNA insertions were homozygous. The *flc-20* mutant contains a modified *Ds* element inserted in the first intron of *FLC* [21].

Plants were grown on MS agar plates in a 16 h light∶8 h dark period under fluorescent light at 22°C. Vernalization was at 4°C.

RNA was extracted using Qiagen Plant RNeasy Mini columns with an on-column DNase treatment according to the manufacturer's protocol. cDNA was synthesized using Superscript III (Invitrogen), primed with oligo dT (distal and proximal COOLAIR transcripts) or with gene specific primers for the *FLC* 5′ unspliced transcript. Quantitative real-time PCR was carried out using an Applied Biosystems 7900HT instrument. Reactions were carried out in a total volume of 10 µl with Platinum Taq DNA polymerase (Invitrogen). Primers used are listed in **Table S1**. PCR were reactions carried out in quadruplicate, quantified using a standard curve of diluted cDNA and normalized to At4g26410 [27].

Chromatin immunoprecipitation was carried out and amplicons for ChIP-qPCR are as described [11]. Primer sequences are given in **Table S1**.

## Supporting Information

Figure S1 **H3K27me3 ChIP qPCR across** ***FLC*** **in C24 and** ***flc-20*** **.** ChIP qPCR for amplicons 1–12 [11] in C24 and *flc-20* plants grown for 12 long days (12LD), 12 long days followed by 4 weeks at 4°C (12+4V) or 12 long days, 4 weeks at 4°C followed by 2 long days (12LD+4V+2LD).
(TIF)

Figure S2 **H3K27me3 ChIP qPCR across** ***FLC*** **in Col** ***FRI*** **, SALK_092716, SALK_140021 and SALK_131491.** ChIP qPCR for amplicons 1–12 (Buzas *et al*, 2011) in Col*FRI*, SALK_092716, SALK_140021 and SALK_131491 plants grown for 12 long days (12LD), 12 long days followed by 4 weeks at 4°C (12+4V), 12 long days, 4 weeks at 4°C followed by 2 long days (12LD+4V+2LD) or 12 long days, 4 weeks at 4°C followed by 5 long days (12LD+4V+5LD).
(TIF)

Table S1 Oligonucleotide sequences.
(XLS)
