# Supplementary figures and images for: Vernalization-Repression of Arabidopsis FLC Requires Promoter Sequences but Not Antisense Transcripts
Source: PLoS One. 2011 Jun 21;6(6):e21513. doi: 10.1371/journal.pone.0021513 (PMC3119698; doi:10.1371/journal.pone.0021513)

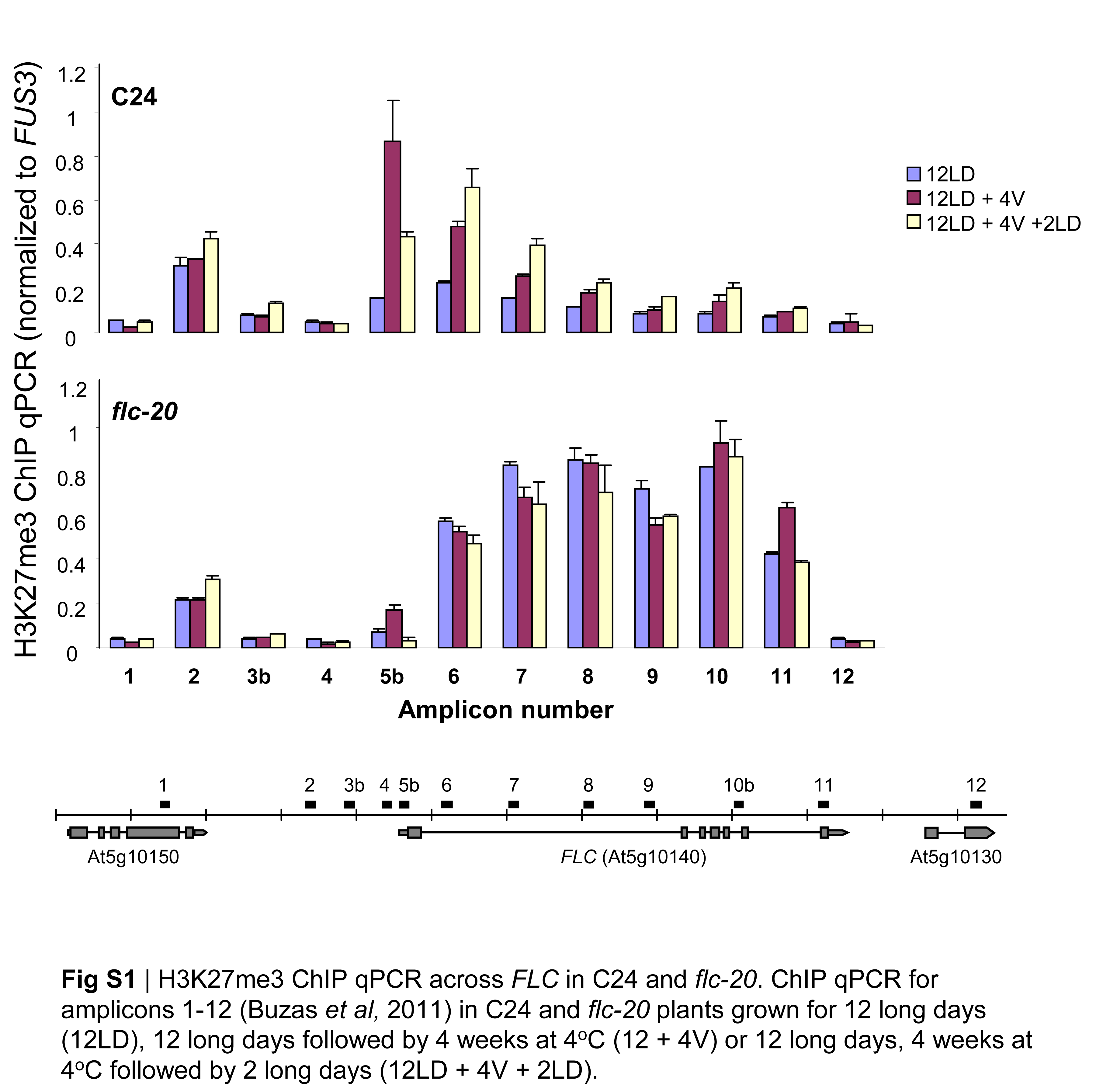

Supplement: Figure S1 — H3K27me3 ChIP qPCR across FLC in C24 and flc-20 . ChIP qPCR for amplicons 1–12 [11] in C24 and flc-20 plants grown for 12 long days (12LD), 12 long days followed by 4 weeks at 4°C (12+4V) or 12 long days, 4 weeks at 4°C followed by 2 long days (12LD+4V+2LD). (TIF) [file pone.0021513.s001.tif]

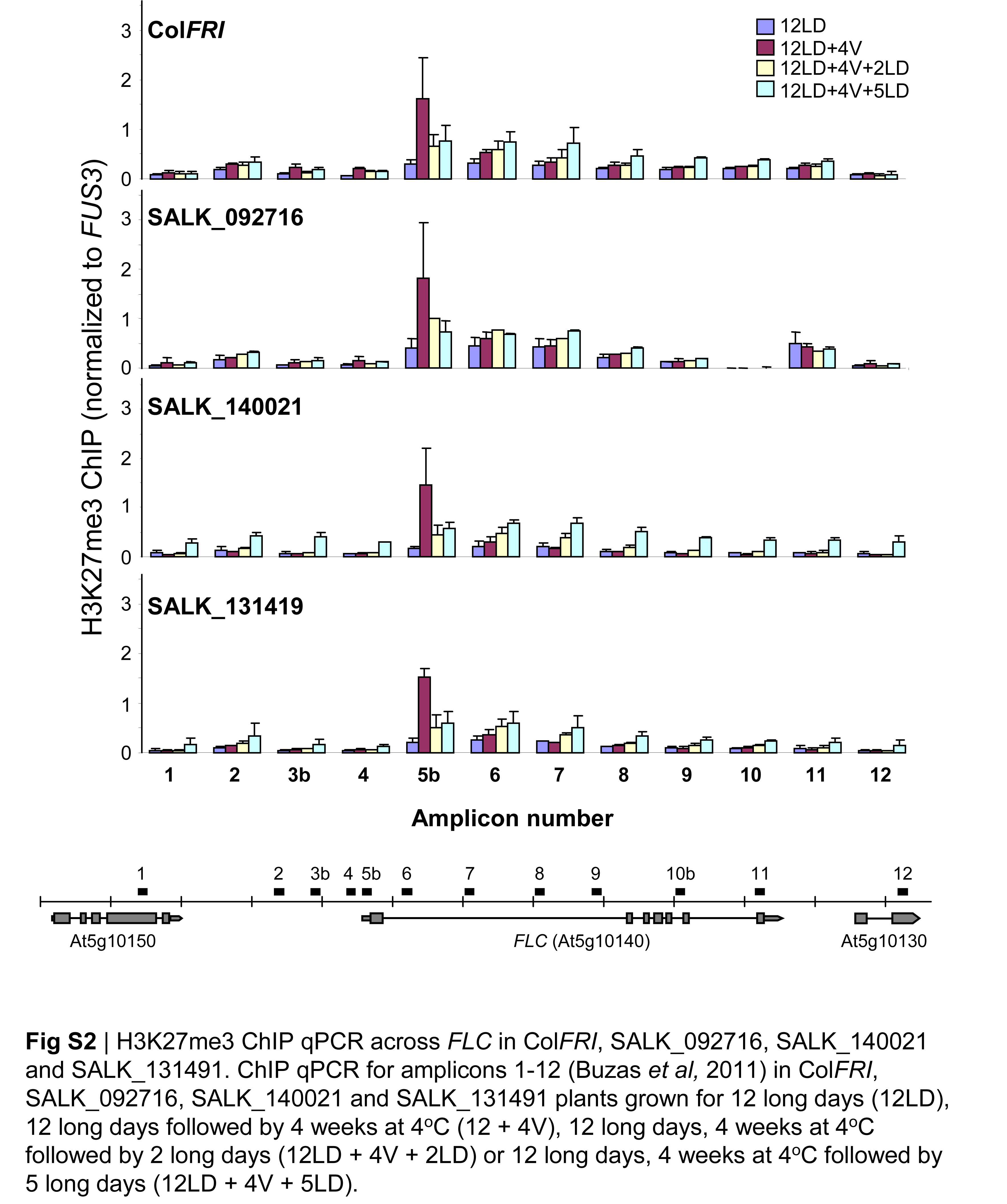

Supplement: Figure S2 — H3K27me3 ChIP qPCR across FLC in Col FRI , SALK_092716, SALK_140021 and SALK_131491. ChIP qPCR for amplicons 1–12 (Buzas et al, 2011) in ColFRI, SALK_092716, SALK_140021 and SALK_131491 plants grown for 12 long days (12LD), 12 long days followed by 4 weeks at 4°C (12+4V), 12 long days, 4 weeks at 4°C followed by 2 long days (12LD+4V+2LD) or 12 long days, 4 weeks at 4°C followed by 5 long days (12LD+4V+5LD). (TIF) [file pone.0021513.s002.tif]
